# Antifungal treatment for invasive Candida infections: a mixed treatment comparison meta-analysis

**DOI:** 10.1186/1476-0711-8-23

**Published:** 2009-06-26

**Authors:** Edward J Mills, Dan Perri, Curtis Cooper, Jean B Nachega, Ping Wu, Imad Tleyjeh, Peter Phillips

**Affiliations:** 1 Faculty of Health Sciences, Simon Fraser University, Burnaby, Canada; 2 Department of Clinical Epidemiology & Biostatistics, McMaster University, Hamilton, Canada; 3 Department of Medicine, McMaster University, Hamilton, Canada; 4 Division of Infectious Diseases, Ottawa Hospital, University of Ottawa, Ottawa, Canada; 5 Departments of Epidemiology and International Health, Johns Hopkins Bloomberg School of Public Health, Baltimore, Maryland, USA; 6 Department of Medicine and Centre for Infectious Diseases, Faculty of Health Sciences, Stellenbosch University, Cape Town, South Africa; 7 Division of Infectious Diseases, Department of Medicine, Research Center, King Fahd Medical City, Riyadh, Saudi Arabia; 8 Division of Infectious Diseases, Department of Medicine, Mayo Clinic, Rochester, MN, USA; 9 Division of Infectious Diseases, University of British Columbia, Vancouver, Canada

## Abstract

**Objectives:**

Invasive fungal infections are a major cause of mortality among patients at risk. Treatment guidelines vary on optimal treatment strategies. We aimed to determine the effects of different antifungal therapies on global response rates, mortality and safety.

**Methods:**

We searched independently and in duplicate 10 electronic databases from inception to May 2009. We selected any randomized trial assessing established antifungal therapies for confirmed cases of invasive candidiasis among predominantly adult populations. We performed a meta-analysis and then conducted a Bayesian mixed treatment comparison to differentiate treatment effectiveness. Sensitivity analyses included dosage forms of amphotericin B and fluconazole compared to other azoles.

**Results:**

Our analysis included 11 studies enrolling a total of 965 patients. For our primary analysis of global response rates, we pooled 7 trials comparing azoles to amphotericin B, Relative Risk [RR] 0.87 (95% Confidence Interval [CI], 0.78–0.96, P = 0.007, I^2 ^= 43%, P = 0.09. We also pooled 2 trials of echinocandins versus amphotericin B and found a pooled RR of 1.10 (95% CI, 0.99–1.23, P = 0.08). One study compared anidulafungin to fluconazole and yielded a RR of 1.26 (95% CI, 1.06–1.51) in favor of anidulafungin. We pooled 7 trials assessing azoles versus amphotericin B for all-cause mortality, resulting in a pooled RR of 0.88 (95% CI, 0.74–1.05, P = 0.17, I^2 ^= 0%, P = 0.96). Echinocandins versus amphotericin B (2 trials) for all-cause mortality resulted in a pooled RR of 1.01 (95% CI, 0.84–1.20, P = 0.93). Anidulafungin versus fluconazole resulted in a RR of 0.73 (95% CI, 0.48–1.10, P = 0.34). Our mixed treatment comparison analysis found similar within-class effects across all interventions. Adverse event profiles differed, with amphotericin B exhibiting larger adverse event effects.

**Conclusion:**

Treatment options appear to offer preferential effects on response rates and mortality. When mycologic data are available, therapy should be tailored.

## Introduction

Invasive fungal infections contribute importantly to morbidity and mortality in immunocompromised patients including those with hematologic cancers, recent transplants, autoimmune disorders, and critical illness. The most common fungal pathogen is *Candida*.

For a number of reasons, early and accurate diagnosis of invasive fungal infections is often difficult and patients from these high risk groups may have evidence of disseminated fungal infection at autopsy that was not identified prior to death[1]. Clinical manifestations of invasive fungal infections often occur at a late stage of infection contributing to diagnostic delays and higher case-fatality rates. Immunocompromised patients may not generate detectable antibody responses to specific fungal pathogens[2]. Furthermore, non-invasive antigen detection methods such as the Fungitec G assay for beta-1–3 glucan are not widely available. Finally, culture techniques are not highly sensitive, and invasive diagnostic techniques may be contraindicated or not applicable to the clinical presentation. Consequently, antifungal treatment for confirmed invasive fungal infections is challenging and evaluations of therapeutic interventions are limited[3]. Several choices of antifungal agents exist that differ greatly with respect to both toxicity and cost[4].

There is an ever-growing literature on the use of antifungal agents in patients with candidemia. Previous systematic reviews have not looked at the relative effectiveness of interventions of confirmed infections [4-6]. Using a systematic review of the literature and meta-analytic techniques, we aimed to quantify the effects of antifungal therapy on confirmed systemic fungal infection response rates, associated mortality and safety when reserved for confirmed cases only. Furthermore, we determined differences in treatment effects across interventions using a mixed treatment comparison meta-analysis.

## Methods

### Eligibility criteria

We included any randomized trial of antifungal therapies for confirmed cases of invasive candidiasis among predominantly adult (≥ 18 years of age) populations. We included randomized trials of any duration. Given that head to head evaluations have existed for decades, studies had to compare antifungal therapy to another antifungal therapy (head-to-head evaluations). Studies had to report on any of the following clinically-important outcomes: clinical response, all-cause mortality; fungal-attributable death, and adverse events. We excluded studies only reporting on dose-comparison or dosage form evaluations. As we were interested in disseminated disease, trials focused on single site fungal infections (mucocutaneous, esophageal, dermatologic, meningeal, bladder, or focal chest) were excluded, as were aspergillosis trials, cryptococcosis and endemic mycoses trials. We excluded drugs no longer recommended by Infectious Disease Society of America Guidelines (IDSA) including ketoconazole[7].

### Search strategy

In consultation with a medical librarian, we (DP, EM) established a search strategy (available from corresponding author on request). We searched independently, in duplicate, the following 10 databases (from inception to May 2009): MEDLINE, EMBASE, Cochrane CENTRAL, AMED, CINAHL, TOXNET, Development and Reproductive Toxicology, Hazardous Substances Databank, Psych-info and Web of Science, databases that included the full text of journals (*OVID, ScienceDirect*, and *Ingenta*, including articles in full text from approximately 1700 journals since 1993). Key search words included words addressing the infections: *Fungus, fungal, fungemia, mycosis, candidiasis, candidemia, candida; and words addressing the interventions: antifungal, amphotericin, azoles, triazoles, fluconazole, itraconazole, miconazole, voriconazole, posaconazole, ravuconazole, flucytosine, echinocandins, caspofungin, micafungin, anidulafungin, confirmed*; and finally a word indicating a randomized trial: random* (wildcard). In addition, we searched the bibliographies of published systematic reviews and collected papers. We contacted the authors of trials for study clarifications, where required. Searches were not limited by language, sex or age.

### Study selection

Two investigators (DP, EM) working independently, in duplicate, scanned all abstracts and obtained the full-text reports of records, that indicated or suggested that the study was a randomized trial evaluating antifungal therapy on the outcomes of interest. After obtaining full reports of the candidate trials, in full peer-reviewed publication, the same reviewers independently assessed eligibility from full text papers.

### Data collection

The same 2 reviewers conducted data extraction independently using a standardized pre-piloted form. Reviewers collected information about the antifungal therapy and type of interventions tested, the population studied (age, setting, underlying conditions), the treatment effect on specified outcomes, adverse events, and specific adverse events addressing renal toxicity and liver impairment. Study evaluation included general methodological quality features, including allocation concealment, sequence generation, a description of who was blinded, use of intention-to-treat analysis and proportion of study population lost-to-follow-up. We entered the data into an electronic database such that duplicate entries existed for each study; when the two entries did not match, we resolved differences through discussion and consensus.

### Data analysis

In order to assess inter-rater reliability on inclusion of articles, we calculated the *Phi *statistic, that provides a measure of inter-observer agreement independent of chance[8]. Our primary outcome of interest is response rates. Secondary outcomes include all-cause mortality, fungal-attributable mortality, and adverse events.

We performed two specific analyses. First, we performed a frequentist fixed-effects meta-analysis of study outcomes across classes of drugs, applying a Relative Risk [RR] and appropriate 95% Confidence Intervals [CIs] of outcomes according to the number of events reported in the original studies. In the event of zero outcome events in one arm of a trial, we used the Haldane method and added 0.5 to each arm[9]. Given the varied size of studies, we pooled studies using a fixed effects approach that recognizes the precision of studies and provides greater to weight to larger studies[10]. We calculated the I^2 ^statistic for each analysis as a measure of the proportion of the overall variation that is attributable to between-study heterogeneity[11]. Given the varied interventions, and the consideration that most trials were not no-treatment or placebo controlled, we pooled studies assessing within-class interventions as head-to-head trials. Our first analysis examined drugs within-class and then examined individual drugs using the mixed treatment comparisons. We initially pooled all azole interventions versus all amphotericin B trials for response and conducted a multivariable meta-regression using the unrestricted maximum likelihood method assessing the impact of individual azoles on the overall estimate of effect and the individual delivery methods of amphotericin B on overall estimate[12]. Our regression covariates were chosen *a priori *and included: amphotericin delivery and allocation concealment. Analyses were conducted using StatsDirect and STATA.

For our second analysis, we examined the relative effectiveness of each individual drug using the Lu-Ades fixed effects method for combining direct and indirect evidence in mixed treatment comparisons, a Bayesian approach[13]. We estimated the posterior densities for all unknown parameters using MCMC (Markov chain Monte Carlo) for each model in WinBUGS version 1.4 (Medical Research Council Biostatistics Unit, Cambridge). Each chain used 100,000 iterations with a burn-in of 500, thin of 5, and updates varying between 80 and 110. We used the same seed number (SEED = 314159) for all chains. The choice of burn-in was chosen according to Gelman-Rubin approach[14]. We assessed convergence based on trace plots and time series plots (available upon request). The accuracy of the posterior estimates was done by calculating the Monte Carlo error for each parameter. As a rule of thumb, the Monte Carlo error for each parameter of interest is less than about 5% of the sample standard deviation[15]. All results are reported as posterior means with corresponding 95% credibility intervals (CrIs). Credibility intervals are the Bayesian equivalent of classical confidence intervals.

## Results

**Figure 1:**
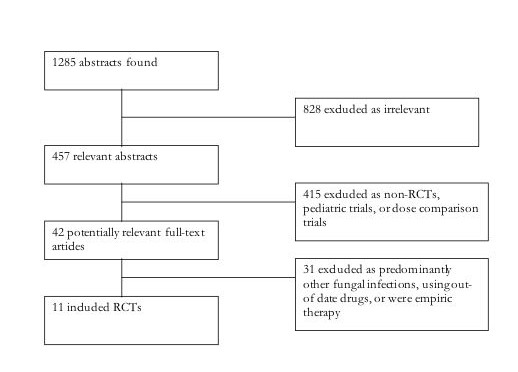
**Flow-diagram of search and included studies**.

Our literature search identified 1284 potentially relevant abstracts of full text articles. Of these, 42 full text RCTs were obtained. We excluded 31, leaving 11 that met our inclusion criteria[2,3,16-24], *Phi *= 0.91, n = 2,554, See Figure 1 and Additional File 1. The majority (n = 7) of studies assessed the head-to-head non-inferiority of azole-class drugs compared to amphotericin B. The azoles include fluconazole[2,3,17,18,23], itraconazole[16], and voriconazole[19]. The other studies assessed anidulafungin to fluconazole[22], micafungin to amphotericin B[20]; caspofungin to amphotericin B[21];and micafungin to caspofungin[24]. Trials were predominantly conducted in populations dominated by patients with hematologic cancers experiencing infection with *Candida *species. The median participant age was 57 years (IQR 56–59).

In keeping with the time periods that the studies were published (1991–2007), reporting of study methodological features was moderate. Seven of eleven trials reported on sequence generation; 6 of 11 on allocation concealment; 3 of 11 on who was blinded; 9 of 11 reported using intent-to-treat principles; and 6 of 11 provided details on participants lost-to-follow-up. An average of 10.8% of study participants were lost-to-follow-up.

### Meta-analysis

#### Global response rates

**Figure 2:**
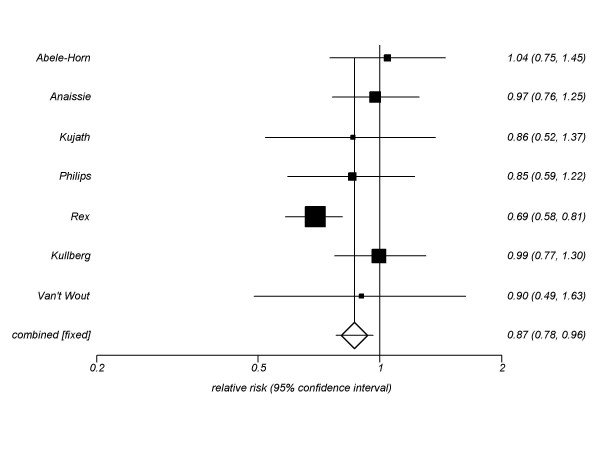
**Global response rate fixed-effects meta-analysis: Triazoles versus amphotericin B**.

For our primary outcome, we pooled 7 trials (n = 965, See Figure 2) assessing azoles to amphotericin B. Our pooled estimate is 0.87 (95% CI, 0.78-0.96, P = 0.007, I^2 ^= 43%, P = 0.09). When we compared only fluconazole trials (5 trials) to amphotericin B, we found similar effects (RR 0.82, 95% CI, 0.74–0.92, P = 0.0009, I^2 ^= 52%, P = 0.07). The itraconazole versus amphotericin B trial (RR 0.90, 95% CI, 0.49–1.63, P = 0.61) and voriconazole versus amphotericin B trial (RR 0.99, 95% CI, 0.77–1.30, P = 0.94) provided similar estimates.

We also pooled 2 trials of echinocandins (micafungin[20] and caspofungin[21]) versus amphotericin B and found a pooled RR of 1.10 (95% CI, 0.99–1.23, P = 0.08). The anidulafungin to fluconazole trial yielded a RR of 1.26 (95% CI, 1.06–1.51, P = 0.001) in favor of anidulafungin[22]; and micafungin to caspofungin (RR 1.00, 95% CI, 0.94–1.08, P = 0.21)[24].

#### All-cause mortality

**Figure 3:**
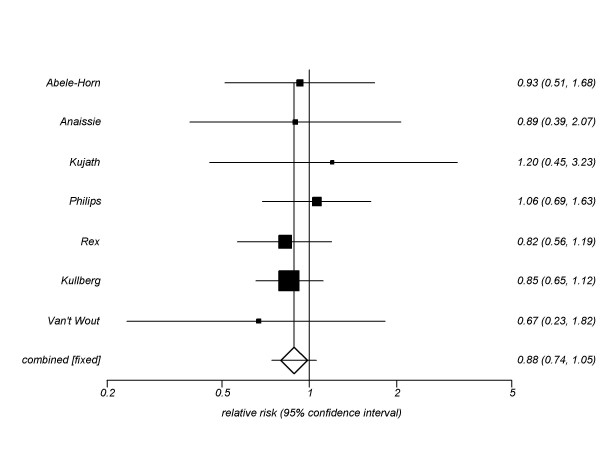
**All-cause mortality fixed-effects meta-analysis: Triazoles versus amphotericin B**.

Our secondary outcomes included all-cause mortality. We pooled 7 trials (n = 965, figure 3) assessing azoles versus amphotericin B for all-cause mortality, resulting in a pooled RR of 0.88 (95% CI, 0.74–1.05, P = 0.17, I^2 ^= 0%, P = 0.96). This was also found when individual azoles were analyzed: fluconazole (5 trials) RR 0.92 (95% CI, 0.73–1.17, P = 0.51, I^2 ^= 0%, P = 0.90; itraconazole (1 trials) RR 0.67, 95% CI, 0.74–1.05, P = 0.20; voriconazole (1 trials) RR 0.85, 95% CI, 0.65–1.12, P = 0.67).

When we assessed echinocandins versus amphotericin B preparations (2 trials) for all-cause mortality, we found a pooled RR of 1.01 (95% CI, 0.84–1.20, P = 0.93). Micafungin versus caspofungin resulted in a RR of 0.85 (95% CI, 0.96–1.11) in the direction of favour of caspofungin. Anidulafungin versus fluconazole resulted in a RR of 0.73 (95% CI, 0.48–1.10, P = 0.34) in the direction of anidulafungin.

#### Fungal-attributable mortality

We also assessed deaths attributable to the fungal infections. When we pooled 5 azole trials versus amphotericin B, we found a pooled RR of 0.84 (95% CI, 0.49–1.42, P = 0.51, I^2 ^= 0%, P = 0.74). When we pooled the 3 echinocandin trials versus amphotericin B, we found a pooled RR of 1.16 (95% CI, 0.75–1.79, P = 0.50). Anidulafungin versus fluconazole yielded a RR of 0.84 (95% CI, 0.48–1.47, P = 0.88).

#### Adverse events

To assess serious adverse events, we pooled 2 trials of azoles versus amphotericin B assessing serious adverse events and found a pooled RR of 0.67 (95% CI, 0.55–0.81, P = <0.0001) in favour of azoles. We also pooled 2 trials of echinocandins versus amphotericin B and found a pooled RR of 0.49 (95% CI, 0.37–0.66, P = <0.0001) in favour of the echinocandins. Micafungin and caspofungin were not dissimilar in their safety profiles (RR 0.94, 95% CI, 0.70–1.29). We found no significant difference between anidulafungin versus fluconazole (RR 0.90, 95% CI, 0.60–1.36, P = 0.66).

When we assessed nephrotoxicity defined variably according to the different studies, we pooled 6 trials of azoles compared to amphotericin B. We found a pooled RR of 0.22 (95% CI, 0.15–0.32, P = <0.0001, I^2 ^= 74%, P = 0.001) in favour of azoles. We also pooled 3 echinocandin compared to amphotericin trials and found a pooled RR 0.31 (95% CI, 0.17–0.57).

Finally, we assessed hepatic enzyme elevations beyond normal. We pooled 3 trials assessing azoles compared to amphotericin B and found a pooled RR of 1.08 (95% CI, 0.79–1.47, P = 0.64, I^2 ^= 0%, P = 0.63). The 2 echinocandin versus amphotericin B trials yielded a pooled RR of 1.03 (95% CI, 0.17–6.26). The single anidulafungin versus fluconazole trial found a RR of 0.21 (95% CI, 0.05–0.83, P = 0.001) in favour of anidulafungin.

#### Mixed treatment comparisons

**Figure 4:**
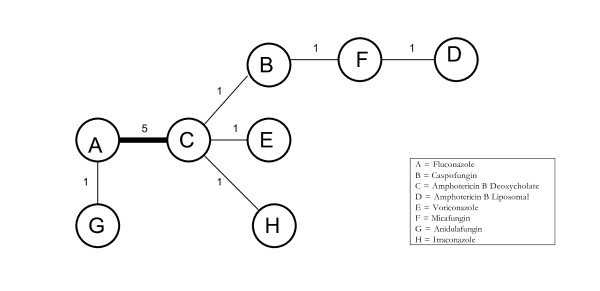
**Network of evidence formed by the eight antifungal treatments compared on the basis of mortality data from 11 studies**. Each treatment is a node in the network. The links between nodes are trials or pairs of trial arms. The numbers along the link lines indicate the number of trials or pairs of trial arms for that link in the network.

**Figure 5:**
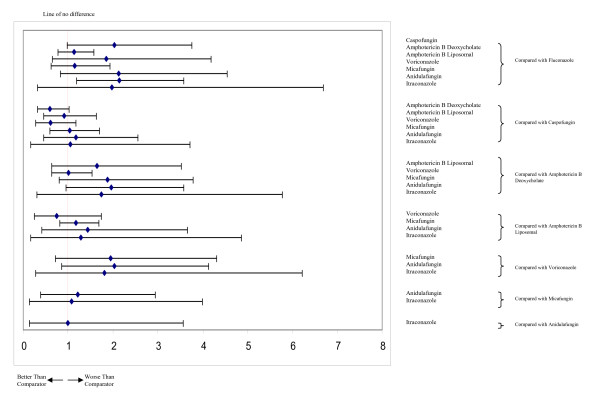
**Caterpillar plots of the odds ratios and 95% CrIs for mixed treatment comparisons, response rates**.

**Figure 6:**
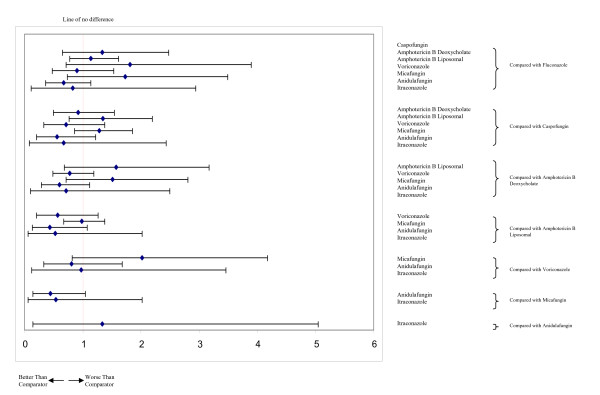
**Caterpillar plots of the odds ratios and 95% CrIs for mixed treatment comparisons, all-cause mortality**.

Figure 4 displays the geometric distribution of the mixed treatment comparison. Figures 5 and 6 display the caterpillar plots. Table 1 reports the odds ratios of response rates for all the pairwise comparisons of the antifungal treatment regimens and table 2 presents estimates of the absolute efficacy for each treatment, along with the estimated probability that each treatment is best (response rate).

**Table 1:** Odds ratios and 95% CrIs for mixed treatment comparisons of confirmed infection studies, Response rates

***Treatment Comparison***	**Odds Ratio**	**95% Credible Interval**
Caspofungin vs. Fluconazole	2.03	(0.98, 3.76)
Amphotericin B Deoxycholate vs. Fluconazole	1.13	(0.78, 1.58)
Amphotericin B Liposomal vs. Fluconazole	1.85	(0.65, 4.19)
Voriconazole vs. Fluconazole	1.14	(0.62, 1.94)
Micafungin vs. Fluconazole	2.13	(0.83, 4.55)
Anidulafungin vs. Fluconazole	2.14	(1.19, 3.58)
Itraconazole vs. Fluconazole	1.97	(0.32, 6.69)

Amphotericin B Deoxycholate vs. Caspofungin	0.60	(0.32, 1.02)
Amphotericin B Liposomal vs. Caspofungin	0.91	(0.45, 1.63)
Voriconazole vs. Caspofungin	0.61	(0.27, 1.18)
Micafungin vs. Caspofungin	1.04	(0.59, 1.70)
Anidulafungin vs. Caspofungin	1.18	(0.45, 2.56)
Itraconazole vs. Caspofungin	1.05	(0.16, 3.72)

Amphotericin B Liposomal vs. Amphotericin B Deoxycholate	1.64	(0.63, 3.52)
Voriconazole vs. Amphotericin B Deoxycholate	1.01	(0.63, 1.54)
Micafungin vs. Amphotericin B Deoxycholate	1.88	(0.80, 3.79)
Anidulafungin vs. Amphotericin B Deoxycholate	1.96	(0.96, 3.58)
Itraconazole vs. Amphotericin B Deoxycholate	1.74	(0.30, 5.77)

Voriconazole vs. Amphotericin B Liposomal	0.75	(0.25, 1.74)
Micafungin vs. Amphotericin B Liposomal	1.18	(0.81, 1.68)
Anidulafungin vs. Amphotericin B Liposomal	1.44	(0.42, 3.66)
Itraconazole vs. Amphotericin B Liposomal	1.28	(0.16, 4.86)

Micafungin vs. Voriconazole	1.95	(0.72, 4.31)
Anidulafungin vs. Voriconazole	2.03	(0.85, 4.13)
Itraconazole vs. Voriconazole	1.81	(0.28, 6.22)

Anidulafungin vs. Micafungin	1.21	(0.38, 2.94)
Itraconazole vs. Micafungin	1.08	(0.14, 3.99)

Itraconazole vs. Anidulafungin	0.99	(0.14, 3.56)

**Table 2:** Absolute treatment efficacy and the probability that each treatment is best in the mixed treatment comparisons analysis using the response data from the confirmed infection studies.

	**Response Rates**	
	**Response %**	**Probability best**

Fluconazole	63.00	0.000

Caspofungin	76.10	0.139

Amphotericin B Deoxycholate	65.40	0.000

Amphotericin B Liposomal	72.98	0.070

Voriconazole	65.03	0.004

Micafungin	75.98	0.200

Anidulafungin	77.49	0.345

Itraconazole	69.33	0.241

Table 3 reports the odds ratios of all-cause mortality for all the pairwise comparisons of the antifungal treatment regimens and table 4 presents estimates of the absolute efficacy for each treatment, along with the estimated probability that each treatment is best (mortality).

**Table 3:** Odds ratios and 95% CrIs for mixed treatment comparisons of confirmed infection studies, all-cause mortality

**Treatment Comparison**	**Odds Ratio**	**95% Credible Interval**
Caspofungin vs. Fluconazole	1.34	(0.65, 2.48)
Amphotericin B Deoxycholate vs. Fluconazole	1.14	(0.78, 1.62)
Amphotericin B Liposomal vs. Fluconazole	1.81	(0.71, 3.90)
Voriconazole vs. Fluconazole	0.90	(0.48, 1.53)
Micafungin vs. Fluconazole	1.73	(0.74, 3.49)
Anidulafungin vs. Fluconazole	0.67	(0.36, 1.14)
Itraconazole vs. Fluconazole	0.83	(0.11, 2.94)

Amphotericin B Deoxycholate vs. Caspofungin	0.92	(0.50, 1.54)
Amphotericin B Liposomal vs. Caspofungin	1.35	(0.77, 2.20)
Voriconazole vs. Caspofungin	0.72	(0.33, 1.38)
Micafungin vs. Caspofungin	1.28	(0.86, 1.85)
Anidulafungin vs. Caspofungin	0.56	(0.21, 1.22)
Itraconazole vs. Caspofungin	0.67	(0.08, 2.44)

Amphotericin B Liposomal vs. Amphotericin B Deoxycholate	1.58	(0.68, 3.17)
Voriconazole vs. Amphotericin B Deoxycholate	0.78	(0.49, 1.19)
Micafungin vs. Amphotericin B Deoxycholate	1.51	(0.72, 2.81)
Anidulafungin vs. Amphotericin B Deoxycholate	0.60	(0.29, 1.12)
Itraconazole vs. Amphotericin B Deoxycholate	0.72	(0.10, 2.50)

Voriconazole vs. Amphotericin B Liposomal	0.57	(0.21, 1.26)
Micafungin vs. Amphotericin B Liposomal	0.98	(0.67, 1.38)
Anidulafungin vs. Amphotericin B Liposomal	0.44	(0.13, 1.08)
Itraconazole vs. Amphotericin B Liposomal	0.53	(0.06, 2.02)

Micafungin vs. Voriconazole	2.02	(0.82, 4.18)
Anidulafungin vs. Voriconazole	0.81	(0.33, 1.68)
Itraconazole vs. Voriconazole	0.97	(0.12, 3.46)

Anidulafungin vs. Micafungin	0.45	(0.15, 1.05)
Itraconazole vs. Micafungin	0.54	(0.06, 2.02)

Itraconazole vs. Anidulafungin	1.34	(0.15, 5.05)

**Table 4:** Absolute treatment efficacy and the probability that each treatment is best for all-cause mortality in the mixed treatment comparisons in confirmed infection studies.

	**Mortality**	
	**Mortality %**	**Probability best**

Fluconazole	28.44	0.006

Caspofungin	33.83	0.008

Amphotericin B Deoxycholate	30.93	0.001

Amphotericin B Liposomal	39.99	0.004

Voriconazole	25.8	0.090

Micafungin	39.16	0.001

Anidulafungin	20.75	0.385

Itraconazole	21.82	0.504

## Discussion

The results of our systematic review and meta-analysis should be of interest to clinicians, policy-makers and patient groups. Our study found similar effects across within-class interventions. Safety profiles indicate that the class of interventions, azoles and echinocandins, offer protection over amphotericin B in terms of adverse events.

There are several important strengths to our meta-analyses that should be considered when interpreting this study. We used extensive searching of electronic databases to identify studies. Thus, we identified more studies than any other systematic reviews[4,5,25]. In order to reduce bias, we conducted our searches independently, in duplicate. We extensively searched the bibliographies of published trials and reviews in order to identify unpublished or obscure papers. Finally, we used methodologically advanced approaches to pool and conducted sensitivity analyses across *a priori *defined covariates.

There are also several limitations to consider when interpreting our analysis. Despite our extensive searching, it is possible that we were unable to identify unpublished trials. Indeed, this issue affects every meta-analysis. We attempted contact through email with 12 study authors to address inclusion and methodological questions but received responses from only 4; a common occurrence with systematic reviews [26]. We examined 3 major clinical outcomes and their sub-categories: global response, mortality and adverse events. It is possible that other outcomes would yield differing effects. We conducted mixed treatment comparisons and demonstrated similar within-class effects of drugs. These comparisons provide compelling comparisons, but only head-to-head trials will provide stronger inferences[27,28].

We considered response according to the original papers' definition of response. We considered response according to the original papers' definition of response. Notably, there is substantial variability in the timing and definitions of response across trials which may limit comparability. Timing of assessments ranged from 7 days after start of therapy[2] to up to 12 weeks after the end of therapy[19], with multiple variations in between. The criteria for response also included a wide variety of clinical and microbiological response definition, as well as consideration of the ability to tolerate randomized therapy[2,21,23]. However, we feel our analysis is useful and as we saw no evidence of discrepancies based on our sensitivity analysis. More similarity in endpoint definitions in future trials would be useful to facilitate across-trial comparisons and evaluation of future therapies.

There has been an ongoing debate over the quality of industry-funded trials of antifungal agents, predominantly in the empiric and prophylaxis trials [29-31]. This contention has predominantly concerned clarifications post-publication, the use of oral amphotericin B compared to intravenous, and the reporting of all-cause mortality compared to cause-specific mortality. In our analysis we have aimed to overcome this discussion through extensive sensitivity analysis, through focusing on confirmed fungal infections, through evaluating relative effectiveness and through presenting both all-cause and cause-specific mortality.

A conundrum in evaluating antifungal therapy is assessing fungle attributable mortality. In most trials, the patients enrolled were at a high risk of mortality due to illness. When we assess all-cause mortality, we recognize that many of the patients would have died from their original disease and not fungal infections. Clinical practice may differ from clinical trial procedures as clinicians may reserve more toxic agents (amphotericin B) for salvage therapy of more severely ill patients who failed short courses of other therapies. However, we could not display a difference between these drug classes when we examined deaths attributable to fungal infections.

We examined the impact of different dosage forms of amphotericin B to determine if their results change and found it did not. A prior systematic review reported a trend towards all-cause mortality benefit and reduction in nephrotoxicity risk in lipid-based formulations of amphotericin B as compared with conventional amphotericin B,[6] however there was no statistically significant difference in efficacy (clinical response) between the dosage formulations. The reasons for discrepancy between all-cause mortality and efficacy were likely a result of clinical and methodologic problems. Dosing and duration of therapy of lipid-based formulations vary widely from study to study, making results difficult to interpret. Trial heterogeneity and small sample size also make it difficult to draw conclusions from comparative studies. An earlier systematic review comparing amphotericin dosage forms found no difference in mortality between lipid and conventional formulations[4]. To our knowledge, no definitive high quality randomized controlled trial comparing amphotericin B dosage formulations has been published since the most recent systematic review.

While we did not include pediatric trials in our analysis, results from a recent systematic review assessing antifungal therapy in children with invasive fungal infections reported congruent conclusions[25]. Although not a meta-analysis, the authors' review (including a supplementation with adult studies) led them to conclude that there was no difference in clinical response between different classes of antifungal agents in the treatment of prolonged febrile neutropenia (empiric therapy) or invasive candidal infection. They did report significant differences in toxicity (particularly nephrotoxicity) between classes that favoured azoles and echinocandins over amphotericin B.

In conclusion, our study suggests that azoles and echinocandins are equally effective interventions for treating invasive candidiasis and confirms the Infectious Disease Society of America (IDSA) guidelines[7], that recommends azoles or echinocandins as the first line treatment for *Candida *infections. Our analysis found similar within-class effects. Amphotericin B offers an effective, but more toxic alternative.

## Competing interests

Curtis Cooper, Ping Wu, Dan Perri, and Imad Tleyjeh declare no conflict of interest. Edward Mills has consulted to Pfizer Ltd., Glaxo-Smithkline, Korean HIRA, and International Society for Clinical Trials. Peter Phillips has been a consultant for Pfizer, Merck Frosst Canada,, and Hoffmann-La Roche; and is a member of the speakers' bureau for Pfizer, Merck Frosst Canada, and Schering-Plough Pharmaceuticals.

## Authors' contributions

EM, DP, PW, CC, IT conceived of the study. EM, DP, PW, CC, IT developed the study protocol. EM, DP, PW, CC, IT conducted the searches and data abstraction. EM, DP, PW, CC, IT, JN, PP analyzed and/or interpreted the data. EM, DP, PW, CC, IT, JN, PP wrote the initial drafts of the manuscript and approved submission. EM, DP, PW, CC, IT, JN, PP revised the manuscript. All authors read and approved the final manuscript.

## Supplementary Material

Additional file 1 **Characteristics of included studies**. Table addressing study populations, interventions and fungal species measured. Click here for file
