# Correction: Antifungal treatment for invasive Candida infections: a mixed treatment comparison meta-analysis

**DOI:** 10.1186/1476-0711-8-25

**Published:** 2009-08-25

**Authors:** Edward J Mills, Dan Peeri, Curtis Cooper, Jean B Nachega, Ping Wu, Imad Tleyjeh, Peter Philips

**Affiliations:** 1 Faculty of Health Sciences, Simon Fraser University, Burnaby, Canada; 2 Department of Clinical Epidemiology & Biostatistics, McMaster University, Hamilton, Canada; 3 Department of Medicine, McMaster University, Hamilton, Canada; 4 Division of Infectious Diseases, Ottawa Hospital, University of Ottawa, Ottawa, Canada; 5 Departments of Epidemiology and International Health, Johns Hopkins Bloomberg School of Public Health, Baltimore, Maryland, USA; 6 Department of Medicine and Centre for Infectious Diseases, Faculty of Health Sciences, Stellenbosch University, Cape Town, South Africa; 7 Division of Infectious Diseases, Department of Medicine, Research Center, King Fahd Medical City, Riyadh, Saudi Arabia; 8 Division of Infectious Diseases, Department of Medicine, Mayo Clinic, Rochester, MN, USA; 9 Division of Infectious Diseases, University of British Columbia, Vancouver, Canada

## Correction

After publication of this work [1], we noted that forest plot labels for figure five should read Worse than Comparator on the left hand side and Better than Comparator on the right. This change has been made in this updated figure (see figure 1).

**Figure 1:**
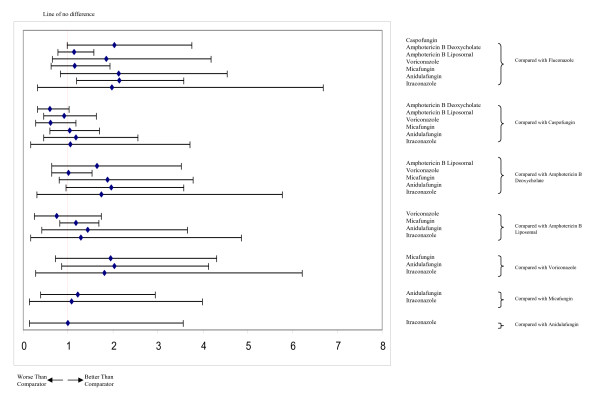
**Caterpillar plots of the odds ratios and 95% CrIs for mixed treatment comparisons, response rates**.

## References

[B1] Mills EJ Perri D Cooper C Nachega JB Wu P Tleyjeh I Phillips P Antifungal treatment for invasive Candida infections: a mixed treatment comparison meta-analysis Ann Clin Microbiol Antimicrob 2009 8 23 10.1186/1476-0711-8-23 19558681 PMC2713200

