# Supplementary material for: Antifungal treatment for invasive Candida infections: a mixed treatment comparison meta-analysis
Source: Ann Clin Microbiol Antimicrob. 2009 Jun 26;8:23. doi: 10.1186/1476-0711-8-23 (PMC2713200; doi:10.1186/1476-0711-8-23)
Supplement: Additional file 1 — Characteristics of included studies. Table addressing study populations, interventions and fungal species measured. [file 1476-0711-8-23-S1.doc]

| Author & year | Definition of response | Population  [Int./Cont.%] | Age  [SD or Range] | | N | | % Neutropenic | | Dose | | Fungal Species  [Int./Cont.%] |
| --- | --- | --- | --- | --- | --- | --- | --- | --- | --- | --- | --- |
|  |  |  | Int. | Cont. | Int. | Cont. | Int. | Cont. | Intervention | Control |  |
| Abele-Horn et al.  1996 | - Resolution of all symptoms and clinical signs with mycological confirmation. | - Non-neotropenic adults (18-80) with evidence of systemic Candida infection - Mixed population, inc:   - Cancer [14/11]   - Gastrointestinal disease [17/22]   - Cardiovascular disease [19/22]   - Respiratory disease [22/19]   - Pancreatitis [14/17]   - Cranial trauma [14/8] | 58.3  [15.0] | 59.7  [11.8] | 36 | 36 | 0 | 0 | Fluconazole  400mg (i.v.) day 1 then 200mg/day | Amphotericin B/5-flucytosine  Combination:  1-1.5mg/kg (70mg max) every other day  Flucytosine:  2.5g 3xdaily | - C. albicans [72/64] - T. glabrata [0/<1] - C. pseudotropicalis [<1/0] - C. tropicalis [<1/<1] - Mixed infections [22/36] |
| Anaissie et al.  1996 | - Defervesence and mycological confirmation of clearance. | - Mixed population, inc:   - Leukaemia and bone marrow transplants [21/15]   - Other cancers [36/48]   - Other disease [43/37] | 62  [22-79] | 58  [18-82] | 75 | 67 | 29 | 31 | Fluconazole  400mg (i.v.) daily for 5 days followed by 400mg orally | Amphotericin B  25-50mg (i.v.) daily for non-neutropenic and 0.67 mg/kg/day for a total dose of 750 mg for neutropenic patients | - C. albicans [57/46] - Non-albicans [33/39] - Undetermined [9/15] |
| Kujath et al.  1993 | - Mycological confirmation of elimination of fungal infections. | - Surgical patients (18+) with systemic candidiasis - Mixed population, inc:   - Peritoneal invasion after gastrointestinal perforation [14/13]*   - Involvement of the lungs and pleura [5/7]*   - Pancreatic necroses [2/2]*   - Esophageal erosion/rupture [1/1]*   - Endocarditis (mitral valve) [0/1]* | 53.0  [17.4] | 55.0  [16.0] | 20 | 20 | n/a | n/a | Fluconazole  400mg day 1 then 300mg daily | Amphotericin B/5-flucytosine  Amphotericin B: 0.1mg/kg day 1, 0.2mg/kg day 2, 0.3mg/kg day 3, up to a max 0.5mg/kg; each administered by perfusor over 6 hours.  Flucytosine:  2.5g 3xdaily | - C. albicans [18/16]* - C. tropicalis [1/2]* - C. glabrata [4/3]* - Candida spp. [2/3]* |
| Rex et al.  1994 | - All signs and symptoms of candida infection are resolved and blood cultures are negative. | - Non-neutropenic patients with blood culture positive for Candida sp. and characteristics demonstrating infection - Population inc:   - Cancer [25/25]   - Renal failure [38/40]   - Diabetes mellitus [15/23] | 58  [2] | 60  [2] | 119 | 118 | 0 | 0 | Fluconazole  400 mg per day | Amphotericin B  0.5 to 0.6 mg/kg per day | - C. albican [68/61] - C. parapsilosis [6/16] - C. tropicalis [17/13] - C. Glabrata [12/14] - C. Krusei [1/2] |
| Phillips et al.  1997 | - Absence of following: i) death within the first 7 days of treatment; ii) evidence of progressive candidal infection; iii) withdrawal from the study. | - Non-neutropenic patients (18+) with candidemia - Mixed population, inc:   - Gastrointestinal disease [56/51]   - Diabetes [30/23]   - Pulmonary disease [34/13]   - Cancer [20/23]   - Renal failure [12/8]   - Cardiovascular disease [4/6]   - Other [22/26] | 65 | 58 | 50 | 53 | 0 | 0 | Fluconazole  800mg (i.v.) loading dose then 400mg daily for 4 weeks; upon clearance of fungemia was administered orally where tolerable; if metastatic infection, treatment extended to 8 weeks. | Amphotericin B  0.6mg/kg (i.v.) once daily to a total dose of 8mg/kg (approx. 2 weeks); if metastatic site(s) of infection then dose increased to total 20mg/kg | - C. albicans [80/62] - C. glabrata [12/13] - C. parapsilosis [6/9] - C. tropicalis [0/4] - C. guilliermondii [0/2] - C. albicans & C. glabrata [2/4] - Saccharomyces or rhodotorula [0/6] |
| van’t Wout et al.  1991 | - At least a 50% decrease in the size of the initial site of severity of the infection or complete clearance. | - Neutropenic patients with proven or suspected fungal infections - Mixed population, inc:   - Leukaemia [81/88]   - Lymphoma [13/6]   - Others [6/6] | 51  [15-74] | 32  [19-51] | 16 | 16 | 100 | 100 | Intraconazole  200mg (oral) every 12 hours | Amphotericin B  0.6mg/kg (i.v.) per day or 0.3mg/kg when in combination with flucytosine (150mg/kg) | Candidiasis   - Definite [19/6] - Probable [-/6] - Possible [19/50]   Aspergillosis   - Definite [6/13] - Probable [44/19] - Unknown [13/6] |
| Kullberg et al.  2005 | - Independent assessment of clinical and mycological clearance at 12 weeks. | - Patients (12+) with candida infection and who were non-neutropenic | 53.6  [13-90] | 53.3  [13-87] | 248 | 122 | 0 | 0 | Voriconazole  6mg/kg (i.v.) every 12 hours for 24 hours then 3mg/kg every 12 hours; after 3 days patients could be switched to 200mg 2 x day (oral) | Amphotericin B followed by fluconazole  0.7-1.0mg/kg per day over 2-6 hours then replaced by 400mg fluconazole (i.v. or oral) per day after a min 3 days and max 7 days | - C. albicans [43/51] - Non-albicans candida species [61/50] - C. tropicalis [21/13] - C. parapsilosis [18/16] - C. glabrata [15/17] - C. krusei [2/1] - Other C. species [6/4] - >2 candida species [5/3] |
| Reboli et al.  2007 | - Global response (clinical and microbiologic) at the end of intravenous therapy in patients who had a positive baseline culture. | - Patients (16+) with defined candidemia or other form of invasive candidiasis - Mixed population, inc:   - Diabetes [35/25]   - Renal failure/insuff [37/36]   - Bacterial sepsis [46/42]   - Neoplastic disease [22/23]   - Disorders requiring transplant [6/4] | 57.0  [17.0] | 59.2  [16.5] | 127 | 118 | 2 | 3 | Anidulafungin  200mg on day 1 then 100 mg daily | Fluconazole  800mg (i.v.) day 1 then 400mg daily | - C. albicans [64/59] - C. glabrata [16/25] - C. parapsilosis [10/14] - C. tropicalis [12/9] - Other C. species [5/3] |
| Mora-Duarte et al.  2002 | - Resolution of all symptoms and signs of candida infection and culture-confirmed eradication (or presumptive eradication for certain nonblood infections). | - Patients (18+) with clinical evidence of infection and with positive cultures from blood or other sterile site - Mixed population, inc:   - Diabetes mellitus [22.9/18.3]   - Active leukaemia or lymphoma [14.7/11.3]   - Renal failure or insufficiency [21.1/26.1]   - HIV infection [3.7/2.6] | 56  [17-84] | 55  [18-81] | 109 | 115 | 12.8 | 8.7 | Caspofungin  70mg (i.v.) loading dose then 50mg per day | Amphotericin B  0.6-0.7mg/kg (i.v.) per day for patients without neutropenia; 0.7-1.0 mg/kg for those with neutropenia | - C. albicans [35.6/54.1] - C. parapsilosis [19.8/18.3] - C. tropicalis [19.8/12.8] - C. glabrata [12.8/9.2] - C. krusei [4.0/0.9] - C. guilliermondii [3.0/0.9] - C. lipolytica [1.0/0] - C. rugosa [1.0/0] - Multiple spp. [3.0/3.7] |
| Kuse et al.  2007 | - Clinical and mycological confirmation of cleared invasive infection. | - Patients (16+) with clinical signs of systemic candida infections and positive cultures from blood or other sterile site - Mixed population, inc:   - Haematological disorder [19/13]   - Acute leukaemia [9/7]   - Solid organ tumour [14/20]   - Diabetes mellitus [12/12]   - Transplant [8/4]   - Non-fungal infection [7/5]   - Gastrointestinal disorders [6/4]   - HIV or other immune disorders [3/6]   - Pancreatitis [3/4]   - Renal failure [3/3]   - Other [9/9] | 54.5  [18-89] | 56.0  [16-97] | 247 | 247 | 13 | 10 | Micafungin  1-hour infusion of 100mg for patients >40kg of weight and 2mg/kg for those <40kg, daily; after 5 days dose potentially increased to 200mg if mycological persistence. | Liposomal amphotericin B  1-hour infusion of 3mg/kg daily; after 5 days dose potentially increased to 5mg/kg if mycological persistence. | - C. albicans [35/30] - Non-albicans candida species [49/43] - C. tropicalis [20/18] - C. parapsilosis [14/12] - C. glabrata [9/6] - C. krusei [2/2] - C. guilliermondii [3/2] - Other [2/5] - Multiple C. spp [6/4] |
| Pappas et al.  2007 | - Clinical and mycological success at the end of blinded intravenous therapy. Clinical success was defined as complete response to treatment (resolution of all symptoms, signs, and abnormal radiographic findings). For patients with candidemia, mycological success was defined as eradication if 2 cultures of blood specimens obtained at least 24h apart had negative results. | - Predominantly nonneoplastic patients   Neutropenia [39/ 11]  Recent surgery [113/76]  Chemotherapy [64/20]  Hemodialysis [43/24]  Diabetes [132/48]  Bacteremia [112/53]  Pancreatitis [22/11]  Renal failure [116/58]  Hepatic failure [10/6]  Stem cell transplant [14/4]  Organ transplant [16/7]  Malignancy [124/52] | 56 (SD 16) | 55 (SD 16) | 390 | 188 | 6.9 | 3.7 | Micafungin 100mg or 150 mg once daily intravenously | Caspofungin 70mg on day one and 50mg per day thereafter | *C. albicans {49/44}*  *N*on–*C. albicans {52/60}*  *C. tropicalis {16/17}*  *C. glabrata {15/17}*  *C. parapsilosis {12/*22}  *C. krusei {4/4}*  Other {6/5} |

**Legend: NA, Not available; int, intervention arm; cont, control arm; %, percentage; i.v., intravenous; *, absolute not percentage.**
